# How to prevent recurrence of atrial fibrillation after catheter ablation

**DOI:** 10.1002/joa3.12974

**Published:** 2023-12-07

**Authors:** Naoya Kataoka, Teruhiko Imamura

**Affiliations:** ^1^ Second Department of Internal Medicine University of Toyama Toyama Japan

**Keywords:** arrhythmia, echocardiography, heart failure, left atrium

To Editor,

Numerous risk factors linked to the recurrence of atrial fibrillation (AF) subsequent to catheter ablation have been posited. Brahier et al., through machine learning analysis, substantiated that an elevated left atrial volume index and premature recurrence stand as independent pre‐procedural determinants for delayed AF recurrence post‐catheter ablation.^1^

In alignment with the authors' findings,^1^ antecedent literature has also delineated the prognostic significance of premature AF recurrence concerning subsequent occurrences at the late phase following catheter ablation.^2^ How do the authors contemplate the clinical ramifications of discerning early AF recurrence from its tardy counterpart? It may warrant considerable attention to categorize any AF recurrence post‐catheter ablation as a primary outcome.

The magnitude of the left atrium is a widely acknowledged risk factor for AF recurrence. Presently, there are no pharmacological modalities available to expedite the reversal of left atrial remodeling. How ought one navigate the management of patients with a substantial left atrium afflicted by AF? Should we exercise prudence in subjecting such cohorts to avoidable catheter ablation? Alternative interventions, like posterior wall isolation, may be preferable in patients with a voluminous left atrium, aiming to diminish its “electrical” dimensions.

In the authors' investigation, both statin and beta‐blocker usage correlated with a heightened incidence of AF recurrence post‐catheter ablation.^1^ Traditionally, these agents exhibit prophylactic efficacy against arrhythmia. Are these agents instigators of AF recurrence, or are they merely potential confounding variables in the context of comorbid dyslipidemia and heart failure?

Pulmonary vein reconnection emerges as a prominent etiology of early AF recurrence, with its prevalence diminishing in the later phases post‐catheter ablation.^3^ Stated differently, non‐pulmonary vein foci assume precedence in the genesis of late AF recurrence. In the study by the authors, early AF recurrence emerged as a significant predictor for subsequent late AF recurrence,^1^ implying a substantial incidence of pulmonary vein reconnection among participants. Augmenting the durability of pulmonary vein isolation emerges as a therapeutic strategy to forestall AF recurrence. Notably, the authors observed no discernible disparity in the prophylactic impact among various sources of ablation energy. Alternative energy sources, such as pulsed‐field ablation, may potentially exhibit greater efficacy in thwarting AF recurrence.

## CONFLICT OF INTEREST STATEMENT

Authors declare no conflict of interests for this article.
